# Point of Contact

**Published:** 1897-10

**Authors:** R. W. Morse

**Affiliations:** Lansing, Mich.


ARTICLE VI.

### POINT OF CONTACT.

BY R. W. MORSE, D. D. S., LANSING, MICH.

Read before the Michigan State Dental Society, June, 1897.

The normal set of teeth in the mouth of a human adult offers sixty points of contact which represents about three-fifths of the probable points at which caries attacks the teeth. Why is it that the teeth decay more at this point than others? It is due to the fact that at this point and just away from it toward the cervical margin, the surface is not self-cleansing, and there becomes attached to the teeth a thin film of agglutinated substance, made up principally of micro-organisms. The food in process of mastication does not disturb them. The brush passes over the buccal and lingual surfaces, and still they remain. The tooth-pick will dislodge some, but it is necessary to use dental floss, or something of that nature to polish and scour them off. If all surfaces of the teeth were kept cleaned of this accumulation, we would soon find that our profession was considerably crowded, but the laity have not been sufficiently drilled on this method, and those who have been instructed do not find the time to do it thoroughly, hence we are called upon to correct the results of their defiance of Providence. This agglutinated mass of organisms secrets acids; at first attacks the cement that binds the enamel rods together, on account of its not being as dense as the enamel rods. Then the rods themselves are decalcified, and gradually disintegrated, leaving an opening into the surface. In such openings as fast as found the microbes establish themselves, and from now on have it all their own way unless the cavities are scientifically stopped by some member of our profession. It is our duty to our patients to instruct them in the use of dental floss and brush to thoroughly cleanse the teeth. Abrasion is the only means by which this can safely be accomplished, and then it is necessary that it should be attended to religiously. Mouth-washes aid in making the microbes uncomfortable but do not remove them. Careful brushing and the use of dental floss in connection with mouth-washes is the best method of attack.

But the thing that I wish to call attention to particularly is the restoration of this point of contact by filling. Dr. Black in his talk before the Chicago society, in February, tells of the new method of cutting away the proximal aspects of the molars and bicuspids that are affected by caries, and replacing with large metallic surfaces properly shaped to control the proximal space, and thus offering a roost for the microbes that they cannot destroy with their acids. I quite agree with the doctor in the main, on the counts he has made. In the case of considerable destruction of the enamel and dentine at this point, but I feel that there is a class of cavities that can be treated in a less heroic manner with good results. The moderate and small-sized cavities around which the enamel is not affected would be of this type. Take two moderate sized proximal cavities as an example in teeth of normal shape and position. At first examination if time permits place between these teeth a pellet of cotton saturated with chloro percha. This is repeated at intervals of three or four days, increasing the size of the pellet until the space is sufficiently large to permit of *thorough* preparation of cavities and of filling and finishing the fillings, and when the teeth resume their natural position the oval surfaces of the fillings should come in contact, and at no point should the enamel on either tooth touch the opposing filling or tooth. The point of contact should be as near the occlusal surface as consistent with the case. Treated in this manner, the margins of the enamel are as self-cleansing as under the best conditions that nature provides and gives the gums in the proximal space good protection. Dr. Miller and Dr. Black have for some time maintained that the fluids of the mouth were responsible for carious teeth, only as they aided or opposed the growth of organism. Dr. Williams has demonstrated this to be the fact, and accounts in this way for predisposition to decay, whether the teeth are what are known as hard or soft teeth. In cases of marked predisposition it is probably necessary to apply the method Dr. Black advocates. With incisive teeth we see many cases of recurrence of decay at the cervical and at the incisive margin of cavities, that were well prepared and carefully filled, but when they resumed their natural position the point of contact was at either of the margins of the enamel rather than the filling. This result probably was due to not having sufficient space by separating to contour the filling sufficiently to allow of their being finished, and still have the contour project beyond the margins and act as a buffer to hold the enamel apart, so that they may be cleansed and cleanse themselves to a degree.

I wish to appeal from the decision and practice, of some good men in the profession that the use of the matrix, is in opposition to these results unless an extreme amount of separation has been procured, while the making and the placing of the filling is a little easier. The completed filling does not often meet the requirement of the restoration of the original tooth.

In crown work where the space is contracted, there is a liability to having straight surfaces in contact, that it is desirable to avoid. Separating will often correct this condition so that cleansing spaces will remain and the gum septum be restored.

In conclusion I wish to appeal to the dental profession to be more thorough in separating teeth to be filled, no matter what filling material is to be used. Make the point of contact of a shape that would best protect the proximal space from the encroachment of food. Have the fillings rest against each other holding the enamel apart from touching adjoining tooth or fillings. This metallic point of contact acts as a protection to the tooth from redecay as a sled runner is protected from wear by the iron shoe fastened upon it.—*Ohio Dental Journal.*

